# Evolution Is a Quantitative Science

**DOI:** 10.1371/journal.pbio.1000381

**Published:** 2010-05-25

**Authors:** John F. Y. Brookfield

**Affiliations:** Institute of Genetics, School of Biology, University of Nottingham, Nottingham, United Kingdom

**Figure:**
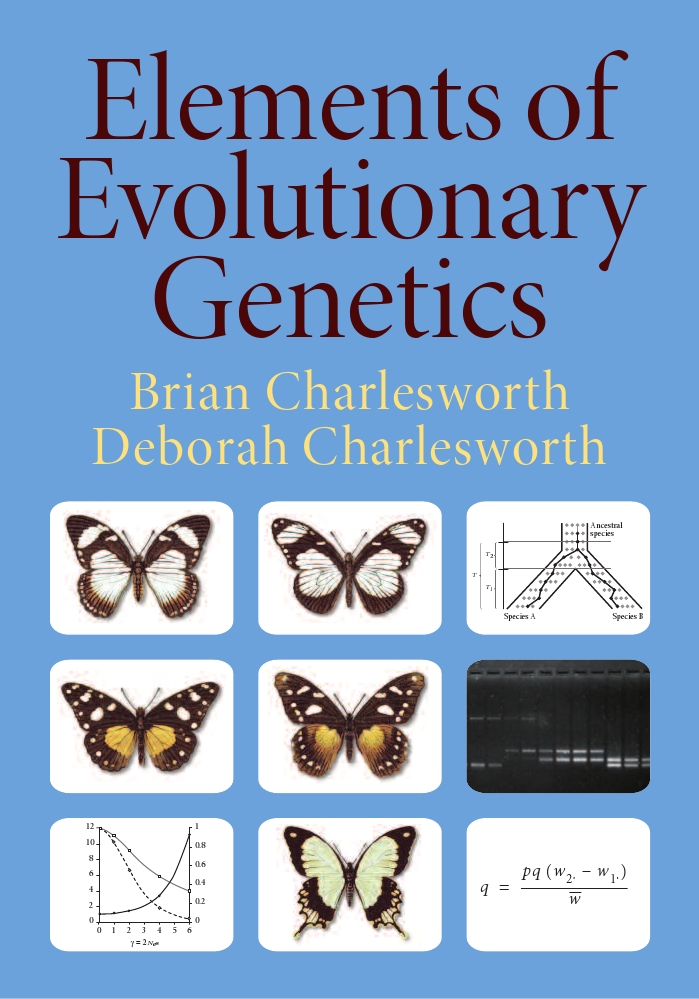
Charlesworth B, Charlesworth D (2010) Elements of evolutionary genetics. Greenwood Village, Colorado: Roberts & Co. 734p ISBN (hardback) 978-0-981-51942-5 US$80.00.

Evolutionary genetics is a mature field of endeavour, and some of biology's greatest minds have contributed to the theory of population genetics. Initially, they faced a problem, in that following the rediscovery of Mendel's results in the early years of the 20th century, some saw an unbridgeable gulf between the sudden changes in appearance seen in the mutant forms of peas studied by Mendel, and the gradual and subtle changes to evolving populations envisaged by Darwin. The so-called Neo-Darwinian synthesis linked these ideas by considering the likely effect of Darwinian natural selection on variations in Mendelian genes, variations which would not necessarily have major effects on organisms' phenotypes. Unusually, for biology, this theory was developed, primarily by Fisher, Haldane and Wright, prior to the existence of data sets to which it could realistically be applied. As a result, the second half of the 20th century saw evolutionary geneticists' struggle to produce data to test theory. They were especially interested in polymorphisms—discrete genetic variations where the rarer type still has an appreciable frequency in the population. So they studied visible polymorphisms, such as the colours and banding patterns of snails, and polymorphisms in the charges of soluble enzymes, until, finally, abundant DNA sequence data became available in the last years of the century.

Now, a resurgence of interest in evolutionary genetics can be predicted, since we will have, through the 1,000 genomes project, for example, data sets detailing population genetic variation genome-wide in many species. We need the tools of evolutionary genetics to describe and explain this variation. What does the variation tell us about population sizes in the past, and rates of gene flow between subpopulations? Which parts of the genome have been subject to purifying and adaptive natural selection, and how strong has this selection been? Recent advances in population genetic theory, in particular, incorporating knowledge that chromosomes include linked sites that are subject to different forces such as selected versus neutral sites, create powerful methods that can help in answering these questions. For these and other reasons, a strong grounding in evolutionary genetics must be included in all bioinformatics and genomics courses. Educators will find that foundation in *Elements of Evolutionary Genetics*, by Brian and Deborah Charlesworth.

This thorough and accurate textbook represents a remarkable achievement. The rigour of the approach is impeccable throughout, and the text makes clear just how many areas of biology, such as sex, genome structure, migration and population variation, and adaptive evolution itself, can be understood only through the application of formal models in which evolutionary processes are considered in a precise way. Most telling, however, is the consistently quantitative approach to data and their interpretation. While a full appreciation of the book will require some mathematical understanding, the steps required are clearly dealt with in appendices, and the reader is helped by problems in each chapter.

Space precludes a full description of such a major work. The focus shies away from the changes to the genetic material over long-term evolution, and methods in building phylogenetic trees, etc., but rather concentrates on evolutionary change at the genetic level over the short term, exploring how mutation, migration, natural selection, and random drift shape the genetic variation within and between populations and how data can give insight into the underlying evolutionary forces that are at play. Following descriptions of the measurement of genetic variation—the theory of quantitative genetics and the theory of population genetics as it can be applied to infinite populations—the sampling effects that create genetic drift and determine levels of neutral variation are described. The expected variation between populations, the consequences and causes of sex and recombination, and the interpretation of genome structure in population genetic terms, all of which are found in the second half of the book, represent areas of particular recent interest. All have been investigated at the theoretical level and much of this theory has been contributed by Brian and Deborah Charlesworth themselves. An example is the expected, and observed, evolution of sex chromosomes. If a single chromosome, such as the mammalian Y chromosome, determines sex, its presence as a sole copy in the cell prevents it from ever undergoing recombination. The lack of recombination will attenuate the power of natural selection to maintain genes on the chromosome, leading to the genetic degeneration so often seen.

But the lessons presented in this text and indeed of evolutionary genetics itself are not restricted to students. In the 21st century there has been increasing emphasis in the need for modelling, testing, refinement and parameter estimation in the biological sciences, as has been captured by the term “systems biology.” However, it is remarkable that many advocates of this approach seem unaware that, in evolutionary genetics, such “predictive biology” has been the standard approach for decades. In the application of a “systems” approach to evolutionary questions, a danger is that a new systems biology community may spend their time reinventing the population genetics wheel.

But why has evolutionary genetics stood apart from biology's resolutely qualitative, rather than quantitative, tradition? Most remarkably, while biomechanics employs the laws of physics, and biochemistry is founded on the quantitative science of chemistry, evolutionary genetics is based on axiomatic foundations that are entirely biological, and yet are capable of precise mathematical formulation. The rules of Mendelian genetics, encapsulated by unbiased inheritance and random mating in a diploid genetic system, predict Hardy-Weinberg frequencies, the binomial sampling of gametes in finite populations determines the properties of genetic drift, and, with a Poisson process of mutation, the complex theory of neutral genetic variation can be established on the basis of very simple assumptions.

However, while the axioms underlying neutral variation are based on the simple biology, the phenotypes, including the Darwinian fitness, of genotypic variants created by mutation have irreducibly complex biological causes, and, for this reason, the incorporation of selected variation into population genetics is more difficult. Consequently, selective theories cannot be as precise as those involving neutrality, so selection, as a potential explanation for a particular data set, cannot easily be pitted against neutrality in a symmetrical Bayesian framework. Rather, neutrality supplies a null hypothesis against which data can be tested, and data showing the signs of selection can then be used as the basis of estimation of selective parameters. But, if I have a criticism of the developments in population genetics that this new volume so admirably describes, it is that some selective models are being created axiomatically with, my guess is, insufficient biological input. An example is the prediction of the distribution of the fitness effects of advantageous new mutations, where this distribution can be derived from Fisher's geometrical argument or, more recently, from the theory of extreme values. So, for example, Fisher's geometrical argument considers a mutation changing the phenotype, where the phenotype is described by *n* different traits. He asks the question whether a random mutation is likely to move the total phenotype in the direction of an optimum phenotype for the environment. It turns out that when the effect of the mutation is vanishingly small, the probability of a net improvement is around half, but this drops rapidly as the size of the mutant's effect increases, with the rate of decrease increasing with increasing *n*. From these simple premises alone can be derived a prediction of the quantitative relationship between the size of the effect of a mutation and the probability that it is advantageous. But I don't find myself confident that this derivation, or indeed one from extreme value theory, contains enough biology to be likely to be correct.

Nevertheless, biologists must get used to the increasingly quantitative approach that this work typifies. Biology is pervaded by the mistaken idea that the formulation of qualitative hypotheses, which can be resolved in a discrete unequivocal way, is the benchmark of incisive scientific thinking. We should embrace the idea that important biological answers truly come in a quantitative form and that parameter estimation from data is as important an activity in biology as it is in the other sciences.

